# The effects of deep neck muscle-specific training versus general exercises on deep neck muscle thickness, pain and disability in patients with chronic non-specific neck pain: protocol for a randomized clinical trial (RCT)

**DOI:** 10.1186/s12891-019-2880-x

**Published:** 2019-11-14

**Authors:** Pegah Kashfi, Noureddin Karimi, Anneli Peolsson, Leila Rahnama

**Affiliations:** 1 Department of Physiotherapy, University of Social Welfare and Rehabilitation Sciences, Tehran, Iran; 2 Department of Medical and Health Sciences, Division of Physiotherapy, Faculty of Health Sciences, Linköping University, Linköping, Sweden, Linköping University, Linköping, Sweden; 3 Department of Physical Therapy, University of North Georgia, Dahlonega, USA

**Keywords:** Neck pain, Exercise, Deep neck muscles, Muscle thickness

## Abstract

**Background:**

Altered thickness, cross-sectional area and activity of deep neck muscles have frequently been reported in patients with chronic non-specific neck pain (CNNP). It is claimed that these muscles do not recover spontaneously. These muscles provide a considerable amount of cervical stability. Therefore, various therapeutic exercises have been recommended to recover from resulting complications. However, most exercise protocols do not target deep neck muscles directly. Thus, this might be a reason for long-lasting complications. Accordingly, the purpose of the present study is to discuss a randomized controlled trial (RCT) protocol in which we aim to investigate and compare the effects of neck-specific exercise programmes versus general exercise programmes in patients with CNNP.

**Methods:**

A 2*2 factorial RCT with before-after design. Sixty-four participants with CNNP will be recruited into the study. They will be randomly divided into two groups, including specific neck exercise and general exercise. Each exercise programme will be carried out three times a week and will last for 8 weeks. Primarily, dorsal and ventral neck muscle thickness, pain and disability and secondarily, muscle strength, quality of life, sleep quality, fear avoidance and neck range of motion will be assessed at the baseline and immediately at the end of the exercise protocol.

**Discussion:**

The results of this study will inform clinicians on which type of exercise is more beneficial for patients with CNNP.

**Trial registration:**

IRCT2017091620787N2, Sep 16 2017.

## Background

Two thirds of the adult population suffer from chronic non-specific neck pain (CNNP) [1], which is associated with disability, activities of daily living (ADL) difficulties, work dissatisfaction, and economic and social costs [2–4]. In addition, altered muscle cross-sectional area, thickness, size, and activity of deep neck muscles have been frequently reported in previous research studies [4–7]. Falla et al. [5] and Kim et al. [6] showed deep neck flexors atrophy and altered electromyography activity (EMG) in patients with neck pain. Rahnama et al. [7] and Fernández-de-las-Peñas et al. [8] demonstrated deep neck extensors muscle atrophy and altered EMG activity following chronic neck pain. These structural and activity changes in deep neck muscles are claimed to be reasons for chronicity and recurrences of the neck pain [9, 10]. To reduce and compensate for negative impacts of such changes, therapeutic exercise is one of the most common and acceptable treatments. Various exercises including neck muscle strengthening, stretching and stabilizing exercises were recommended to overcome these complications in patients with CNNP [11–20]. However, the answer to the question of which exercises are the most effective remained controversial.

Janda suggested that in the presence of pain, neck and back superficial muscles are prone to guarding while the deep muscles are vulnerable to weakening [21]. muscle guarding could be induced by pain. When the pain remains the vicious cycle may lead more muscle guarding [22]. In this regard, stretching exercises may be recommended to reduce neck muscle guard in patients with CNNP [23, 24]. Additionally, general neck exercises are believed to improve general fitness and physiological interactions [17]. On the other hand, deep neck flexor training regimes have increased deep neck flexor thickness and strength in patients with CNNP. Landén Ludvigsson et al. [25] and Peolsson et al. [26] studied different exercise regimes in individuals with chronic whiplash associated disorder (WAD) and found more psychologic and clinical benefits from specific neck exercise (SNE) compared to general neck exercise (GNE). Deep cervical muscle thickness and the effects of exercise on their atrophy were not assessed [25, 26]. However, considering the efficacy of both mentioned exercise regimes, no study has yet compared the effects of these two exercise approaches on CNNP. Furthermore, despite the important role of deep neck extensor muscles in providing neck stability and healthy function [27], their training has been missed in many studies. Therefore, prescribing exercises targeting deep neck extensor muscles is essential as it is claimed that their recovery following pain inhibition would not happen automatically [6, 7].

General neck exercises are commonly prescribed by clinicians in routine management of CNNP based in order to improve general muscle activity and function and reduce muscle guarding. However, to the best of our knowledge, there are not yet any studies comparing the effects of general neck exercises and specific exercises targeting deep neck muscles (both flexor and extensor muscles) in CNNP patients. Therefore, the aim of the present clinical trial is to evaluate and compare the effects of two exercise programmes including GNE and SNE on muscle morphology, pain, disability and functional measures in CNNP patients.

### Study hypotheses

Primary hypothesis: There would be significant improvements in pain, disability and deep cervical muscle thicknesses in both groups, but changes would be more prominent in the SNE group compared to the GNE group in people with CNNP.

Secondary hypothesis: There would be significant improvements in cervical active range of motions, maximum voluntary isometric contraction, sleep quality, quality of life and fear avoidance in both groups, but changes would be more prominent in the SNE group compared to the GNE group in people with CNNP.

## Methods

### Trial design

The trial utilizes a single blinded design and conforms to the SPIRIT guidelines (Additional file 1).

### Study setting

This study will be conducted at the University of Social Welfare and Rehabilitation Sciences’ physiotherapy Research Lab and has been registered in the Iranian Registry of Clinical Trials (WHO subgroup) with the clinical trial registry number IRCT2017091620787N2. The project is in accordance with the ethical principles and national norms and standards approved by the University of Social Welfare and Rehabilitation Sciences, Tehran, Iran (IR.USWR.REC.1396.194).

### Participants

A total of 64 participants of both sexes (women and men) aged 18–55 years with CNNP will be recruited from the main universities in Tehran province by advertising and placing posters on the universities’ bulletin boards. Those individuals who respond to the advertisements will be interviewed for eligibility by the researcher. The researcher is the physiotherapist who measures the study outcomes. Participants will be included if they give their signed, written informed consent. Other inclusion criteria include: 1) BMI ≤ 25 [28], 2) unilateral neck pain [29], 3) current neck pain (sense of pain anywhere in posterior of cervical spine, from superior nuchal line to the first thoracic spinous process) [30] of at least 3 months’ duration in the past year [31], 3) pain intensity greater than 30 mm on the visual analogue scale (VAS) [5], and 5) diagnosed with CNNP. Neck pain was defined as having pain on the posterior aspect of the cervical spine anywhere from nuchal line to the first thoracic spine [30]. Volunteers will be excluded if they report acute neck pain, history of any spinal surgery and disc disease, cervical fracture or tumour, radicular pain into their shoulders or any positive neurologic signs, history of cervical trauma or crash injury, congenital abnormality of the spine, inflammatory diseases, vertigo or vestibular disorders [18, 31, 32].

### Procedure

**Fig. 1:**
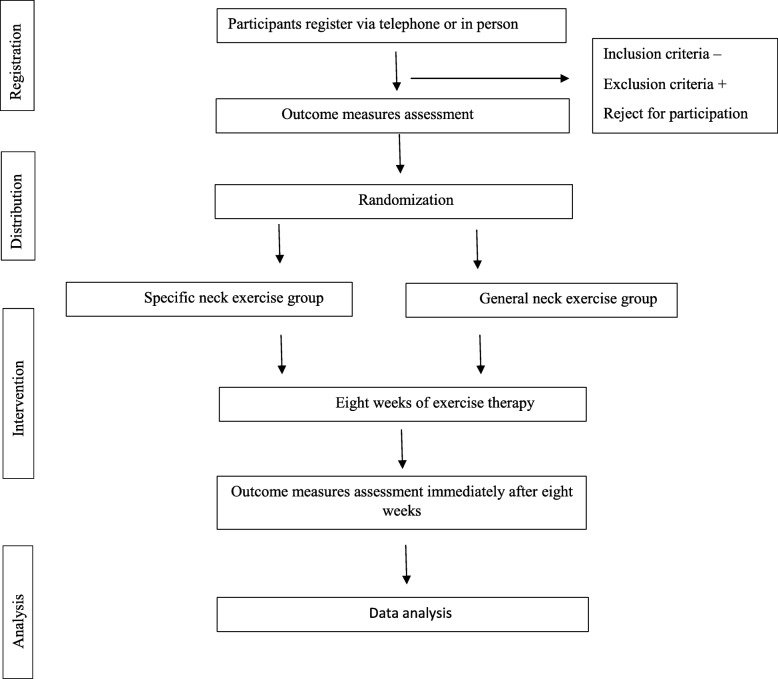
The diagram demonstrating randomized controlled trial protocol

All necessary information about the trial including the study purpose and procedure will be given to the participants both orally and in writing. The participants will then be allocated randomly to two exercise groups, the SNE and GNE groups. Randomization will be performed using sealed envelopes. None of the participants will be aware of the other training group. Both exercise programmes will continue for 8 weeks (3 days per week with three sets on each day and five repetitions in each set). One set will be supervised by a physiotherapist at the university physiotherapy clinic and two other sets will be carried out at home by the participant himself/herself [26, 33]. Before beginning the study, after baseline measurements and randomization, each participant will be familiarized with his or her own exercise programme. They will be taught how to perform their exercises and will be monitored to ensure that they carry out them correctly. Each participant will receive a pamphlet explaining all exercises (SNE or GNE) using schematic pictures. The primary and secondary outcome measures will be assessed before and after 8 weeks of intervention, except for pain which will be assessed daily (Fig. 1).

The participants will be advised not to use other forms of treatments during the trial. However, they will be asked to notify the researcher at every session if they use analgesic medication in an unavoidable situation.

### Reliability

In order to assess the repeatability level of measurements, a primary study will be conducted on 10 participants with measuring dorsal and ventral muscles thicknesses, neck active range of motions and maximum voluntary isometric contraction (MVIC). After 3–7 days, the participants will be asked to return and the measurement process will be carried out again. After the second assessment, participants will start training as previously described. Interclass coefficient correlation (ICC) and standard error of measurements (SEM) will be reported.

### Assessor

The study will be conducted by a physiotherapist with 2 years of clinical practice who has been trained for ultrasound imaging for 6 months. The physiotherapist will assess and record outcome measures and will supervise one of the three sets of exercises to make sure that participants perform the exercises correctly (three times per week). The other two sets of exercises will be performed by the participants without the physiotherapist’s supervision. The participants will be asked to notify the trial physiotherapist if they feel any discomfort when carrying out the exercises. The test-retest reliability of the trial physiotherapist in measuring the outcome measures will be assessed before beginning the study procedures on two separate days, 3 days apart.

### Intervention programmes

The exercise period will be performed for 8 weeks, 3 days per week, three sets each day with five repetitions in each set. The final goal is to increase the exercise difficulty to 20 repetitions in each set [26]. The exercise difficulty will be increased by two repetitions per week considering participants’ tolerance. If increasing the exercise repetition causes participants to feel pain, the exercise repetition is therefore beyond their tolerance and the repetition number will not change for the next week [26]. There will be no specific exercise order in either group.

#### Specific neck exercise group (SNE)

The participant will lie on the experimental bed in a supine position with bent knees and relaxed hands laid beside him or her on the bed. A thin layer of a towel will be placed under the participant’s head to keep his or her head and neck in the neutral position (forehead and chin should be parallel to the ceiling) [26, 33] (Table 1).

**Table 1:** Details of specific neck exercises

I.	Participants move their eyes upward and backward without any movement of the head and neck and hold for 5 s.
II.	Participants move their eyes downward and forward without any movement of the head and neck and hold for 5 s.
III.	Participants do chin talk by bringing their chin closer to their sternum, and hold for 5 s.
IV.	Participants perform a light isometric nodding accompanied by downward movement of their eyes. They will be asked to apply moderate resistance to their chin with their own hands in the opposite direction of the chin movement.
V.	Participants press the occiput area (behind the head) with submaximal pressure to the bed and hold for 5 s.

#### General neck exercises

All exercises will be performed in the standing position (the participant will stand relaxed while looking forward with the head and neck in a neutral position), except for press-up exercises which will be performed in a sitting position (feet on the ground, hands on the armrest of a chair). A 1 kg weight will be added to shoulder shrug exercise from week 4 to the end of the programme [13, 15, 26] (Table 2).

**Table 2:** Details of general neck exercises

I.	Participants move their heads slowly up and down without holding at the end ranges.
II.	Participants rotate their heads to the right and left slowly (to see their shoulders), without holding at the end ranges.
III.	Participants bend their heads to the side, bringing their ears close to their right and left shoulders. No holding at the end ranges.
IV.	Participants abduct their shoulders by bringing their arms into the frontal plane.
V.	Participants flex their shoulders by bringing their arms into the sagittal plane.
VI.	Participants hold their elbows in 90° of flexion and their forearms in pronation. They then move their hands towards and away from their trunks.
VII.	Participants try to bring their shoulders up as close to their ears as possible. They then lower their shoulders without holding at the end ranges. (Shoulder shrugs.)
VIII.	Participants bring their body up on their extended elbows. They then lower their body without holding at the end range.

### Outcome measures

#### Primary outcome measures

##### Pain

The visual analogue scale (VAS) is a 100 mm valid and reliable scale for recording pain with ICC = 0.96 to 0.98 according to a previous study [34]. The number 0 on this scale means no pain and 100 means the worst imaginable pain. The participants will be asked to show their painful areas on the posterior region of their necks using their hands. They will be instructed to show us if their pain is on their left and right upper cervical, lower cervical, and the trapezius. The participants’ current pain will be measured before and after 8 weeks of intervention. In addition, pain intensity will be measured at each intervention session before and after performing exercises [34].

##### Disability

The Iranian version of neck disability index (NDI) questionnaire (ICC = 0.90–0.97) [35] will be used to determine the participants’ disability. This index contains ten items including questions about activities of daily living (seven items), pain (two items), and concentration (one item). Each question is scored from zero to five. The NDI scores will be presented as a percentage of the maximum score, in which 0% indicates no disability and 100% indicates maximum disability [36].

##### Cervical muscle thickness

The thicknesses of dorsal neck muscles including trapezius, splenius capitis, semispinalis capitis, semispinalis cervicis, and multifidus and ventral neck muscles including longus colli and sternocleidomastoid will be measured on the painful side using an ultrasound device (Ultrasonix ES 500) with linear array, 45 mm, and 6.6 MHz probe for dorsal muscles and 12 MHz probe for ventral muscles. According to established ultrasonographic studies, Ultrasound is a valid and reliable device to measure dorsal (ICC = 0.98–0.99) [37] and ventral (ICC = 0.98–0.99) neck muscles thickness [38]. Muscle thickness will be recorded at rest and during maximum voluntary isometric contraction (MVIC).

#### Dorsal neck muscle imaging

Participants will be asked to sit on the experimental chair with their head and neck in a neutral position, with their hands at rest on their legs and their feet on the ground [39]. The assessor then palpates the neck to find the fourth cervical vertebral (C4) spinous process [39]. The probe will be placed on C4 transversely, and will then be moved slightly towards the painful side to see the echogenic vertebral lamina clearly [39]. At this level the measurement will be taken from the muscle’s surrounding fascia which are the superior and inferior fascia at the widest distance. at rest and during a 10 s MVIC. While participants keep the pressure constant, the ultrasound image will be frozen for thickness measurement. The procedure will be repeated three times and the mean thicknesses will be used for data analyses to reduce measurement errors [39–41].

#### Ventral neck muscle imaging

Longus colli and sternocleidomastoid muscles thicknesses will be measured while participants lie supine with bent knees and their hands resting on the bed. It is essential that participants’ heads and necks are in a neutral position. To achieve this, a thin layer of a towel will be put under participants’ occiputs in order to ensure that their foreheads are parallel to the ceiling [42]. The assessor will then place the probe 2 cm below the Adam’s apple and move it about 1 cm laterally towards the painful side to observe the muscle, carotid artery and thyroid cartilage [42]. The muscle thicknesses will be measured at rest and during a 10 s MVIC. To record the flexor muscle thicknesses during contraction, a pressure biofeedback will be placed under participants’ occiputs [12]. Participants will be asked to nod, holding the nod until the pressure unit shows 30 mmHg and then hold it for 10 s. While participants keep the pressure constant, the ultrasound image will be frozen for thickness measurement. The procedure will be repeated three times to reduce measurement errors [42].

### Secondary outcome measures

#### Neck active range of motion (AROM)

Cervical AROM in flexion, extension, rotation to the right and the left and lateral flexion to the right and the left will be measured with a universal goniometer. Goniometric assessment of neck AROM is a reliable technique with ICC ranged from 0.83 to 0.98 [43]. To measure flexion and extension AROM, the centre of the goniometer will be placed over the external auditory meatus, the stationary arm will be perpendicular to the ground and the moving arm will be aligned parallel to the longitudinal axis of the nose. To measure the lateral flexion AROM, the centre of the goniometer will be placed over the spinous process of the seventh cervical vertebra, the stationary arm will be perpendicular to the ground (in the direction of the thoracic vertebral spinous process) and the moving arm will be aligned to the dorsal head midline (the line passing the occipital protuberance). To assess rotational AROM, the centre of the goniometer will be placed over the centre of the cranial aspect of head, the stationary arm will be parallel to an imaginary line passing between the two acromial processes, and the moving arm will be aligned with the tip of the nose. First, the assessor will show the movements to participants and instruct them to perform them correctly so that they do not use their thoracic vertebra. The participants will then be asked to move their heads in three anatomical planes in six directions so that the assessor can measure their neck AROM [44].

#### Neck muscle maximum voluntary isometric contraction (MVIC)

A tensiometer will be used to record neck extension and flexion MVICs. Measuring MVIC using dynamometry is a reliable technique with ICC = 0.94 [45]. The tensiometer has two bands, one fixed to a wall and the other one placed around the participants’ heads [45]. Participants will be asked to sit on a chair with their feet on ground and their arms resting on their thighs. To record neck extension MVIC, they will be instructed to push their head backwards without any movements in their heads and trunks [46]. To record neck flexor MVIC, participants will turn back to the tensiometer and the tensiometer band will be placed on the participants’ foreheads. Participants will then be instructed to push their foreheads towards the band. All MVIC measurements will be repeated three times and the maximum MVIC will be recorded for further analyses. There is a 30 s rest between each MVIC performance and the physiotherapist will lead the patient orally by pushing during the task [47, 48].

#### Sleep quality

The Iranian version of the Pittsburgh Sleep Quality Index (PSQI) questionnaire with the reported ICC equal to 0.77 [49], will be used to assess participants’ sleep quality. The PSQI is a self-rating questionnaire with 19 questions in seven categories: sleep quality, sleep latency, sleep duration, habitual sleep efficiency, sleep disturbances, use of sleeping medication, and daytime dysfunction. Each component is rated from zero to three. Higher scores indicate poorer sleep quality [49].

#### Quality of life

The Iranian cultural comparable version of the short-form 36 (SF-36) questionnaire with ICC equal to 0.70 [50] will be used to assess participants’ quality of life. This questionnaire contains 36 questions in eight dimensions of quality of life, including physical functioning (ten questions), role limitations due to physical health problems (four questions), social functioning (two questions), bodily pain (two questions), general mental health (five questions), vitality (four questions), role limitations due to emotional health (three questions), general health perceptions (five questions) and reported health transition (one question) [51].

#### Fear avoidance

The Iranian version of the Tampa scale questionnaire with ICC larger than 0.80 [52] will be used to investigate participants’ fear of movement. This questionnaire contains 17 questions, each of which are scored from 1 to 4. Total scores range from 17 to 68, with the higher scores indicating stronger fear avoidance beliefs [52].

### Randomization and allocation concealment

The participants will be allocated randomly to one of the two training groups: SNE and GNE. Simple randomization with sealed envelopes in which one of the letters A or B is written will be used for group allocation. Each participant will choose one of the sealed envelopes to be allocated to one of the exercise groups. The envelope will then be returned to the envelope box The randomization will be carried by a physiotherapist who is independent of the study. The allocation concealment will be revealed after the final measurement.

### Sample size

The sample size estimates are based on relevant studies (SD_1_ = 0.32, SD_2_ = 0.56) and the mean difference of 1.1 cm for deep cervical muscle thickness changes [53].We accept the significance level of 5% and the power equal to 80%. Accordingly, 32 participants have been calculated to be recruited in each group [53].

### Trial status

Among 64 recruited participants of this study, 56 participants have completed their exercise programs and eight participants is still performing their exercise programs. The estimated final day of the trial is 18 the March 2019.

### Statistical analysis

SPSS version 24 will be used for statistical analyses. Intra class correlation of coefficient (ICC) and standard error of measurement (SEM) will be used to assess the repeatability level of measurements. The distribution-based technique, in which the constant score with the standard deviation or effect size is compared, will be used to determine the minimal clinically important difference (MCID). The Kolmogorov-Smirnov test will be used to compare the study sample with reference probability distribution. In order to compare the groups per-protocol analyses will be performed. Mixed ANOVA will be used to investigate the main and interaction effects of within and between subject factors on outcome measures. Correlation analysis will be used to assess the possible associations between the variables so that we can investigate any effect of each intervention program on the strength of the evaluated correlations. In order to make the results comparable, the effect size, mean differences, and their confidence intervals will be reported.

## Discussion

Neck pain is a common musculoskeletal problem, and has episodic and periodic types which cause ADL and work difficulties, disability and economic and social costs for both patients and society [2–4]. Therefore, introducing the most effective treatment protocol would seem to be essential in order to decrease not only the pain but also the complications which are not spontaneously reversible. This trial seeks to be more precise about the types of training and investigates whether a specific training that targets deep muscles has any superiority to general exercises for the neck. Based on present evidence, specific exercise training is effective and general training has positive effects on clinical symptoms. However, to the best of our knowledge, no study has yet examined the effects of specific exercises targeting the deep neck muscles, including deep neck extensor and flexor muscles, in patients with chronic neck pain. The results of this trial are expected to increase the efficacy of prescriptive exercise training and also to bring about improvements in individuals with CNNP conditions.

## Supplementary information

**Additional file 1:** SPIRIT 2013 Checklist: Recommended items to address in a clinical trial protocol and related documents*.

## Data Availability

The data set will be available on reasonable request to the corresponding author.

## Abbreviations

AROM: Active range of motion
CNNP: Chronic non-specific neck pain
MVIC: Maximum voluntary isometric contraction
SD: Standard deviation
SPIRIT: Standard Protocol Items: Recommendations for Interventional Trials
SPSS: Statistical Package for the Social Sciences
VAS: Visual analogue scale
